# Granger causality in integrated GC–MS and LC–MS metabolomics data reveals the interface of primary and secondary metabolism

**DOI:** 10.1007/s11306-012-0470-0

**Published:** 2012-10-25

**Authors:** Hannes Doerfler, David Lyon, Thomas Nägele, Xiaoliang Sun, Lena Fragner, Franz Hadacek, Volker Egelhofer, Wolfram Weckwerth

**Affiliations:** 1 Department of Molecular Systems Biology, University of Vienna, Althanstrasse 14, 1090 Vienna, Austria; 2 Department of Chemical Ecology and Ecosystem Research, University of Vienna, Vienna, Austria

**Keywords:** Plant systems biology, Metabolomics, Cold acclimation, Granger causality, Mass spectrometry, Differential Jacobian

## Abstract

*Metabolomics* has emerged as a key technique of modern life sciences in recent years. Two major techniques for metabolomics in the last 10 years are gas chromatography coupled to mass spectrometry (GC–MS) and liquid chromatography coupled to mass spectrometry (LC–MS). Each platform has a specific performance detecting subsets of metabolites. GC–MS in combination with derivatisation has a preference for small polar metabolites covering primary metabolism. In contrast, reversed phase LC–MS covers large hydrophobic metabolites predominant in secondary metabolism. Here, we present an integrative metabolomics platform providing a mean to reveal the interaction of primary and secondary metabolism in plants and other organisms. The strategy combines GC–MS and LC–MS analysis of the same sample, a novel alignment tool MetMAX and a statistical toolbox COVAIN for data integration and linkage of Granger Causality with metabolic modelling. For metabolic modelling we have implemented the combined GC–LC–MS metabolomics data covariance matrix and a stoichiometric matrix of the underlying biochemical reaction network. The changes in biochemical regulation are expressed as differential Jacobian matrices. Applying the Granger causality, a subset of secondary metabolites was detected with significant correlations to primary metabolites such as sugars and amino acids. These metabolic subsets were compiled into a stoichiometric matrix N. Using N the inverse calculation of a differential Jacobian J from metabolomics data was possible. Key points of regulation at the interface of primary and secondary metabolism were identified.

**Electronic supplementary material:**

The online version of this article (doi:10.1007/s11306-012-0470-0) contains supplementary material, which is available to authorized users.

## Introduction

The interaction of primary and secondary metabolism in plants and other organisms is probably one of the most active regulatory circuits balancing biotic and abiotic environmental pressures to the system. Secondary metabolites therefore serve as important functional units to cope with these stresses and at the same time provide the richest resource of natural products in medicine and nutrition. Besides their obvious interconnectivity, in most metabolomics studies either primary or secondary metabolites are analysed to reveal the metabolic response of the system to a specific perturbation. However, by analysing complex reprogramming of metabolism in response to environmental changes it becomes clear that a comprehensive interpretation is hardly possible without integration of the data as recently shown by combining GC–MS and LC–MS metabolomics data in a long-term biodiversity experiment (Scherling et al. 2010). In another study metabolic cross-talk during the final ripening process in melon fruit (*Cucumis melo*) was revealed by the identification of large metabolic association networks and global patterns of coordinated compositional changes of primary and secondary metabolism (Moing et al. 2011). However, due to the complexity of interactions between various pathways it is hardly possible to unambiguously trace back changes in metabolism to regulatory cues. The study of such complex interactions is focused by the research field of systems biology attempting to resolve the relationship between individual entities, for example molecules or genes, in a complex system in order to understand the resulting system behaviour. Numerous experimental and mathematical approaches to comprehensively analyse plant metabolic networks have been proposed relying on iterative processes of model development, model simulation and experimental validation (Giersch 2000; Morgan and Rhodes 2002; Rios-Estepa and Lange 2007; Nägele et al. 2010). In addition to approaches of mathematical modelling, systems biology also comprises multidimensional data analysis focusing on interpretation of the results of experiments on transcriptomics, proteomics and metabolomics (Weckwerth 2011a). Recently, we developed a toolbox, called COVAIN, which provides statistical methods allowing for the comprehensive analysis of high-dimensional metabolomics data (Sun and Weckwerth 2012). The Granger causality analysis, which is amongst other methods also implemented in COVAIN, is a time-series correlation analysis, which allows for the identification of variables being controlled by time-lagged values of other variables. This method originates from the investigation of causal relations within econometric models (Granger 1969), and recently it was also applied in a study of yeast metabolism (Walther et al. 2010). Granger causality analysis considers the time-series of variable X and Y, which can be expressed as follows (Eq. 1):

1 $$ \begin{gathered} X(t) = \sum\limits_{i = 1}^{d} {C_{X,i} X(t - i)} + \sum\limits_{i = 1}^{d} {C_{XY,i} Y(t - i) + } R_{X} (t) \hfill \\ Y(t) = \sum\limits_{i = 1}^{d} {C_{YX,i} X(t - i)} + \sum\limits_{i = 1}^{d} {C_{Y,i} Y(t - i) + } R_{Y} (t) \hfill \\ \end{gathered} $$

C_X,i_ is the regression coefficient between X(t) and X(t−i), and C_XY,i_ is the regression coefficient between X(t) and Y(t−i). X(t) and Y(t) represent the conditions at time point t, R is the residual error, and d is the maximal time lag between the variables. An association between X and Y is assumed to exist if the *p* value of the F test on the cross-coefficients is less than 0.01 (Sun and Weckwerth 2012). Hence, Granger causality between variables may be identified if a time series of variables is available which shows a dynamical behaviour and allows for the robust estimation of regression coefficients. Besides this pair-wise analysis of variables, Granger causality is also applicable to more than two variables using a Granger model of the n-th order (Granger 1969).

Each single point of a time series at which variables are determined describes a quasi steady state of the considered system such as the metabolite contents describe the metabolism of a plant leaf cell at a certain time point. A so-called Jacobian matrix characterizes the local dynamics around such a steady state. In this context, the dynamic representation of a metabolic pathway can be described by a system of differential equations where changes of metabolite concentrations over time are expressed as functions of all metabolite concentrations considered within the system. The corresponding Jacobian is the matrix of all first-order partial derivatives of all functions on all metabolites. Hence, the Jacobian describes the influence of the change of each metabolite upon the changes of other metabolites.

Applying an approach that links the Jacobian with the covariance of the involved metabolite concentrations (Steuer et al. 2003; Weckwerth 2011b, 2003), statistical features of the data are being connected to dynamical properties of the system (Eq. 2):

2 $$ JC + CJ^{T} = - 2D $$

Here, C is the covariance matrix of metabolites, J is the Jacobian and D represents a fluctuation matrix taking into account the apparent stochasticity of the data. If the stoichiometric matrix N of the underlying metabolic system is exploited this equation can be used for inverse calculation of the Jacobian from metabolomics covariance data (Weckwerth 2011b). As it was described previously (Sun and Weckwerth 2012), the solution of J cannot be obtained directly due to under-determined equations. To circumvent this problem, reversibility and irreversibility of the reactions within a metabolic network are integrated in the “directed stoichiometric matrix” and non-zero entries of J can be calculated (Sun and Weckwerth 2012). In cases when J contains less non-zero entries than C, an over-determined problem exists, which can be solved, e.g. by minimizing total least squares.

To reveal perturbation sites between two different metabolic states we recently introduced the differential Jacobian matrix (Sun and Weckwerth 2012). The differential Jacobian matrix, *dJ*_*ij*_, is defined by the relative change between the Jacobian matrices a and b, representing two metabolic states (Eq. 3):

3 $$ dJ_{ij} = \text{log}_{2} \left( {abs\left( {\frac{{J_{a,ij}
}}{{J_{b,ij} }}} \right)} \right) $$

The entries of the differential Jacobian describe the relative changes between Jacobian a and b for every element ij.

Summarizing both methods of Granger causality analysis and the differential Jacobian, it becomes obvious that neither statistical correlation analysis nor mathematical modelling of metabolic networks is capable of providing a comprehensive functional interpretation on their own. This is due to the fact that knowledge of metabolite interaction is needed for model development while unknown interactions can only be estimated by statistical methods like correlation analysis. On the other hand, statistical correlation analysis does not provide adequate tools for far-reaching analysis of metabolite interaction as they are represented by enzymatic interconversions. To overcome this limitation, we developed an approach for integrated analysis of primary and secondary metabolism in *Arabidopsis thaliana* during exposure to low temperature from the same sample by combined use of GC–MS and LC–MS techniques. Merging methods of correlation analysis and mathematical modelling indicated key points of regulation at the interface of primary and secondary metabolism during cold exposure in *A. thaliana*. For the first time, the inverse calculation of a differential biochemical Jacobian from metabolomics data is demonstrated.

## Materials and methods

### Chemicals

Methanol (HPLC-grade), Chloroform (anhydrous, >99 %, p.a.), Acetonitrile (UHPLC-grade) and Pyridine (anhydrous, >99,8 %) were purchased from Sigma-Aldrich (Vienna, Austria). Formic acid (98–100 %) was purchased from Merck (Vienna, Austria). *N*-methyl-*N*-(trimethylsilyl) trifluoroacetamide (95–100 %) was purchased from Macherey–Nagel (Düren, Germany). Chloramphenicol (>98 %) and Ampicillin trihydrate (analytical standard) were purchased from Fluka (Vienna, Austria). ^13^C_6_-Sorbitol (99 %) was purchased by Campro Scientific (Berlin, Germany).

### Plant material and harvest

*Arabidopsis thaliana* plants Col-0 (wild type) were cultivated in a growth chamber under controlled conditions. The substrate for plant growth was composed of Einheitserde® ED63 and perlite. Plants were fertilized once with NPK fertilization solution (WUXAL®Super; MANNA°-Dünger, Ammerbuch, Germany). Light intensity was 250 μmol m^−2^ s^−1^ for 8 h followed by 16 h darkness, relative humidity was 60 % with a temperature of 22 °C. Of 120 *A. thaliana* specimen, 12 plants were harvested in a non cold acclimated state directly from the growth chamber; the remaining plants were put to 4 °C with the same light intensity and humidity applied as described above. Every 48 h, 2 h after the onset of the light period the plants were harvested randomly resulting in a total number of ten time points including time point “0” of the non cold acclimated state. Leaves were sampled in three biological replicates, representing pools of four plants each. Immediately after cutting leaves from the plants, they were put in aluminium bags and quenched in liquid nitrogen. Plant material was ground to a fine powder using mortar and pestle with liquid nitrogen. Sample material was stored at −80 °C between all steps until extraction.

### Extraction procedure and sample preparation for primary and secondary metabolite analysis

For GC–MS analysis a protocol according to Weckwerth et al. was used (Weckwerth et al. 2004). Deep frozen plant material was ground to a fine powder using a mortar and pestle under constant adding of liquid nitrogen. About 45 mg of each replicate was transferred to pre-cooled reaction tubes. For the extraction process, 1 ml of ice cold extraction mixture (methanol:chloroform:water, 5:2:1, v:v:v) was subsequently added. Additionally, 10 μl of internal ^13^C_6_-Sorbitol standard were added into each tube. Tubes were vortexed for several seconds and incubated on ice for 8 min to achieve a good extraction. Hereupon, the samples were centrifuged for 4 min at 14,000×*g*, separating the soluble compounds from remaining cell structure components. For phase separation, the supernatant was then carried over into a new tube containing 500 μl deionized water and 200 μl chloroform. After 2 min of centrifugation at 14,000×*g*, the water/methanol phase, containing the polar metabolites, was separated from the subjacent chloroform phase and completely dried out overnight.

Samples were derivatised by dissolving the dried pellet in 20 μl of a 40 mg methoxyamine hydrochloride per 1 ml pyridine solution and incubation on a thermoshaker at 30 °C for 90 min. After adding of 80 μL of *N*-methyl-*N*-trimethylsilyltrifluoroacetamid (MSTFA), the mixture was again incubated at 37 °C for 30 min with strong shaking.

A solution of even-numbered alkanes (Decane C10, Dodecane C12, Tetradecane C14, Hexadecane C16, Octadecane C18, Eicosane C20, Docosane C22, Tetracosane C24, Hexacosane C26, Octacosane C28, Triacontane C30, Dotriacontane C32, Tetratriacontane C34, Hexatriacontane C36, Octatriacontane C38, Tetracontane C40) was spiked into the derivatized sample before GC–MS analysis in order to infer the retention time and create the retention index.

For LC–MS analysis, frozen plant leaf material was ground as for GC–MS sample preparation, followed by addition of 1 ml pre-chilled 80/20 v:v MeOH/H_2_O extraction solution containing each 1 μg of the internal standards Ampicillin and Chloramphenicol per 50 mg of fresh weight. Samples were hereupon centrifuged at 15,000×*g* for 15 min and the supernatant was placed into a new tube and completely dried out overnight. The resulting pellet was then dissolved in 100 μl of a 50/50 v:v MeOH/H_2_O solution and centrifuged again for 15 min at 20,000×*g*. The remaining supernatant was then filtered through a STAGE tip (Empore/Disk C18, diameter 47 mm) into a vial with a micro insert tip. Before analysis lipid components were removed by adding 500 µl of chloroform, centrifugation and separation of the non-polar-phase to avoid contamination of the ESI ion transfer capillary.

### GC-TOF/MS analysis

GC–MS measurements were carried out on an Agilent 6890 gas chromatograph coupled to a LECO Pegasus® 4D GCxGC-TOF mass spectrometer. Injection was performed splitless with a 4 mm inner diameter tapered liner containing deactivated glass wool at an injection temperature of 230 °C. Components were separated on an Agilent HP-5MS column (30 m length, 0.25 mm diameter, 0.25 μm film). The initial oven temperature was 70 °C hold for one minute, followed by a ramp of 9 °C per minute with 310 °C target temperature, which was held for 5 min, followed by a 20 °C jump to 330 °C, also being held for 5 min. Data acquisition rate on the mass spectrometer was 15 spectra/second with a detector voltage of 1600 V. Length of a run was 35 min, after an acquisition delay of 7.5 min, and the mass range was 40–600 *m*/*z*. The obtained raw data were processed by the on-board LECO ChromaTOF® software capable of spectrum deconvolution, base line correction and automated peak searching. Compounds were manually annotated with the help of a retention index and mass spectra comparison to a spectra library (Kopka et al. 2005) and, with a minimum match factor of 850, arranged into a reference table (Supplement 1). Subsequently, chromatograms acquired under the same conditions were matched against the reference compound list. For relative quantification, peak areas from selected unique fragment ions for every identified compound were used. The obtained data matrix was directly exported from the Pegasus® software into an Excel worksheet.

### LC–MS metabolomics

5 μL of sample were injected on a Waters HSST3 column (100 mm length × 100 μm I.D.) using a nano LC ultra 1D pump from Eksigent and a HTC PAL autosampler. Mobile phase A consisted of H_2_O with 0,1 % formic acid (FA) and mobile phase B of 90 % acetonitrile (ACN) with 0, 1 % FA. A constant flow rate of 500 nl/min was used with the following nonlinear gradient:

**Table:** 

Time (min)	% A	% B
0	95	5
3	95	5
20	85	15
22	83	17
27	80	20
42	68	32
57	54	46
68	25	75
72	5	95
90	5	95
95	95	5
115	95	5

A nano ESI source from Thermo Scientific was used. All data were acquired in positive ionisation mode. Each Full Scan, resolution 60,000, was followed by a data dependent MS^2^ scan, resolution 7,500, of the most abundant ion, which was subjected to Collision Induced Dissociation (CID) using a normalized collision energy of 50 %. The Orbitrap was used to acquire MS spectra, ranging from 100 to 2,000 *m*/*z*, as well as MS/MS spectra. Only recognized charge states were allowed to trigger MS^2^ spectra generation. Non-peptidic precursor selection was enabled, and the dynamic exclusion list was set to 500 values, with a duration of 90 s, and a repeat count of one. Minimum signal threshold was set to 1,000 (absolute value). The temperature of the heated capillary and electrospray voltage were 180 °C and 2 kV, respectively. After MS analysis, mzXML files were created using MassMatrix MS Data File Conversion (v3.9c) from raw files and analyzed using the MetMAX algorithm which is based on PROTMAX (v2.7) (Hoehenwarter et al. 2008) with the following settings: Ion Count, Intensity, Decimals: two cut, all charge states, Environment 10 min, Unite neighbours, Intensity expected one, no retention time filter. Results from this data extraction process were arranged in an Excel file of the same order as the GC–MS obtained data.

### Statistical data analysis

GC–MS as well as LC–MS obtained data were normalized to fresh weight and internal standards (^13^C_6_-Sorbitol for GC–MS, Ampicillin for LC–MS). To reduce the high variation in the LC–MS data, the data set was filtered as follows: the coefficient of variation (CV) of each time point was calculated, as well as the average CV of all ten time points. All values equal or lower than 30 % were disregarded. Data matrices from both measurements were combined. Data pre-processing, principal component analysis (PCA), Granger causality analysis as well as calculation of the differential Jacobian matrices were performed in the Matlab® Toolbox COVAIN (Sun and Weckwerth 2012). After filling of missing values and adjustment of outliers, as well as log10 and* z*-transformation, Granger analysis was applied. Granger parameters were set to time lag 1 with a significance *p* value of 0.05. The Granger causality analysis was performed pair-wise on metabolite concentrations applying linear regression.

## Results and discussion

### Cold-induced reprogramming of primary metabolism in *Arabidopsis thaliana*

Primary metabolite content of leaf tissue of *A. thaliana*, accession Columbia (Col-0), were identified by GC-TOF/MS before and after 2, 4, 6, 8, 10, 12, 14, 16 and 18 days of exposure to 4 °C. Fast, intermediate and slow steps of metabolic readjustment could be distinguished and results are explicitly summarized in Supplement 2. To shortly summarize the findings, components of primary carbohydrate metabolism displayed a fast increase due to cold exposure. Besides the increase of carbohydrate contents, the fastest response to cold exposure was the significant decrease of aromatic amino acids, phenylalanine, tyrosine and tryptophan. Significant accumulation of organic acids, like ascorbic acid, citrate or malic acid, were observed after 4 to 8 days of cold exposure. Accumulation of pyruvic acid was found to be part of late metabolic readjustment after 12 days at 4 °C. Changes in polyamine content were not unique, as putrescine accumulated within the first 2 days of cold exposure while spermidine accumulated significantly after 12 days.

### Analysis of the interaction between primary and secondary metabolism

**Fig. 1:**
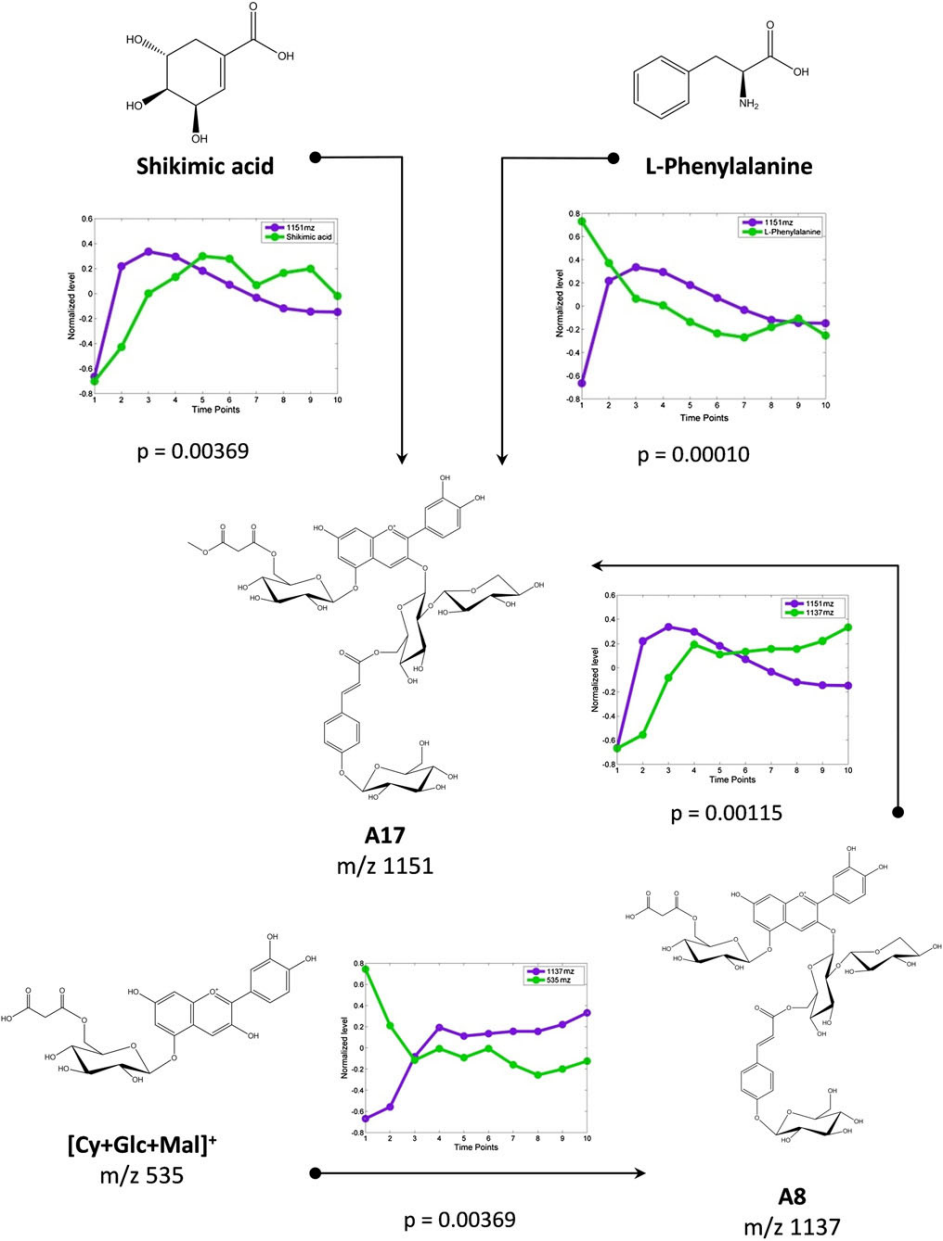
Granger causality analysis between molecules of GC–MS and LC–MS measurements from primary and secondary metabolism. Key metabolites for phenylpropanoid synthesis identified by GC-TOF/MS from *Arabidopsis* leaves have Granger correlations with compound A17 (Shi and Xie 2010) obtained by LC–MS, either by upregulation (shikimic acid) or downregulation (phenylalanine) over 18 days of cold stress. Also, *m*/*z* 1137, A8, is shown to be a precursor for its methyl ester form, A17, while itself being caused by *m*/*z* 535, Cyanidin 5-O-(6′′′-O-malonyl)glucoside. Corresponding *p* values are depicted in the figure

Applying the alignment tool MetMAX to metabolomics data, which was developed on the basis of the PROTMAX algorithm (Hoehenwarter et al. 2008), and using *ion count* and *intensity* as quantitative parameters in the algorithm to correctly bin the *m*/*z* ratios, about 3,000 *m*/*z* ratios were acquired by each chromatographic run (explicit description of settings are provided in Materials and methods). From this data set 349 *m*/*z* values in total were filtered and reliably identified for all time points of cold exposure by statistical analysis and calculation of the coefficient of variance (CV) (Hoehenwarter et al. 2008). In a next step this LC–MS data set was integrated with the GC–MS data set from the same samples using a function in COVAIN for data integration (Sun and Weckwerth 2012). The interactions between primary and secondary metabolites were investigated by Granger causality analysis—another function of COVAIN. With a *p* value <0.05 approximately 15,000 Granger causations were determined, which were either describing time series correlation between metabolites within the GC–MS data matrix, within the LC–MS data matrix or between components of these two matrices. The results are summarized in Supplement 3. As a consequence of the experimental design, which was intended to stimulate flavonoid accumulation due to low temperature and elevated light intensity, Granger causations were predominantly detected in flavonoid biosynthesis. (Fig. 1). Putative metabolic interaction sites were identified revealing the synthesis of the molecule A17 (*m*/*z* 1151) from shikimic acid and phenylalanine (Fig. 1). Additionally, precursor molecules of A17 could be identified within the LC–MS data set allowing for the reconstruction of the synthesis pathway: the molecule [Cy + Glc + Mal]^+^ (*m*/*z* 535) is substrate for synthesis of A8 (*m*/*z* 1137) which is subsequently methylated to molecule A17 (*m*/*z* 1151). In addition to molecule A17, most cyanidin derivatives were putatively identified to be associated to molecule A1 (*m*/*z* 743) which was previously termed cyanidin 3-O-[2′′-O-(xylosyl)glucoside] 5-O-glucoside (Tohge et al. 2005). Accurate mass-to-charge-ratios of metabolites were used for calculation of sum formulas and putative identifications were confirmed by comparison with existing literature, as well as tracking the specific MS^n^ fragmentation patterns, which are described for flavonoids (Matsuda et al. 2009; Waridel et al. 2001) (Table 1).

**Table 1:** Associated molecules to A1 (*m*/*z* 743) identified by Granger causality analysis

Molecule abbreviation	Mass-to-charge ratio (*m*/*z*)	Name of molecule	Reference	Experimental mass deviation (ppm)	CAS-no.
A2	829.2034 [M + H^+^]^+^	Cyanidin 3-O-[2′′-O-(xylosyl)glucoside]5-O-(6′′′-O-malonyl)glucoside	Tohge et al. 2005	1.0854	866259-91-8
A3	889.2307 [M + H^+^]^+^	Cyanidin 3-O-[2′′-O-(xylosyl) 6′′-O-(*p*-coumaroyl)glucoside]5-O-glucoside	Tohge et al. 2005	−0.5623	866259-92-9
A6	1051.2926 [M + H^+^]^+^	Cyanidin 3-O-[2′′-O-(xylosyl)-6′′-O-(*p*-O-(glucosyl)-*p*-coumaroyl)glucoside]5-O-glucoside	Tohge et al. 2005	0.5707	906811-94-7
A7	1095.2977 [M + H^+^]^+^	Cyanidin 3-O-[2′′-O-(2′′′-O-(sinapoyl) xylosyl)6′′-O-(*p*-coumaroyl)glucoside]5-O-glucoside	Tohge et al. 2005	−0.2739	866259-94-1
A8	1137.2930 [M + H^+^]^+^	Cyanidin 3-O-[2′′-O-(xylosyl)6′′-O-(*p*-O-(glucosyl)*p*-coumaroyl)glucoside]5-O-[6′′′-O-(malonyl)glucoside]	Tohge et al. 2005	−0.4396	475163-06-5
A9	1181.2981 [M + H^+^]^+^	Cyanidin 3-O-[2′′-O-(2′′′-O-(sinapoyl)xylosyl)6′′-O-(*p*-O-coumaroyl)glucoside]5-O-[6′′′′-O-(malonyl) glucoside]	Tohge et al. 2005	−0.4233	864155-73-7
A10	1257.3530 [M + H^+^]^+^	Cyanidin 3-O-[2′′-O-(2′′′-O-(sinapoyl) xylosyl)6′′-O-(*p*-O-(glucosyl)*p*-coumaroyl) glucoside]5-O-glucoside	Tohge et al. 2005	1.5906	n.a.
A11	1343.3509 [M + H^+^]^+^	Cyanidin 3-O-[2′′-O-(6′′′-O-(sinapoyl) xylosyl)6′′-O-(*p*-O-(glucosyl)-*p*-coumaroyl)glucoside]5-O-(6′′′′-O-malonyl)glucoside	Tohge et al. 2005	−0.8188	475163-04-3
A17	1151.3086 [M + H^+^]^+^	Cyanidin 3-O-[2′′-O-(xylosyl)6′′-O-(*p*-O-(glucosyl)*p*-coumaroyl)glucoside] 5-O-[6′′′-O-(methyl-malonyl)glucoside]	Shi and Xie 2010	0.7817	n.a.

### Analysis of cold-induced metabolic perturbation sites—calculation of a differential Jacobian from metabolomics data

**Fig. 2:**
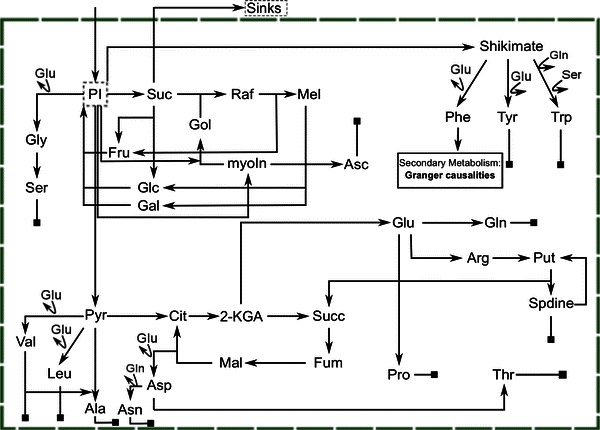
Schematic representation of the primary metabolism in leaf cells of *A. thaliana*. Secondary metabolites identified by Granger causalities are exemplarily integrated derived from phenylalanine. *PI* phosphorylated intermediates, *Glu* glutamate, *Gln* glutamine, *Gly* Glycine, *Ser* serine, *Suc* sucrose, *Fru* fructose, *Glc* glucose, *Gol* galactinol, *Raf* raffinose, *Mel* melibiose, *myoIn* myo-Inositol, *Asc* ascorbic acid, *Gal* galactose, *Phe* phenylalanine, *Tyr* tyrosine, *Trp* tryptophan, *Pyr* pyruvic acid, *Val* valine, *Leu* leucine, *Ala* alanine, *Cit* citric acid, *2-KGA* 2-ketoglutaric acid, *Succ* succinic acid, *Fum* fumaric acid, *Mal* malic acid, *Asp* aspartic acid, *Asn* asparagine, *Arg* arginine, *Put* putrescine, *Spdine* spermidine, *Pro* proline, *Thr* threonine

Based on the data set derived from GC–MS analysis a simplified metabolic network structure was derived comprising interconversions of primary metabolism. The prominent interaction with secondary metabolism via phenylalanine, which was identified by Granger causation, was also included in the network structure (Fig. 2). We focused on the phenylalanine-derived synthesis of putative flavonoids because this is one of the most prominent examples of interaction described in the literature (Winkel-Shirley 2002; Tzin et al. 2012).

**Fig. 3:**
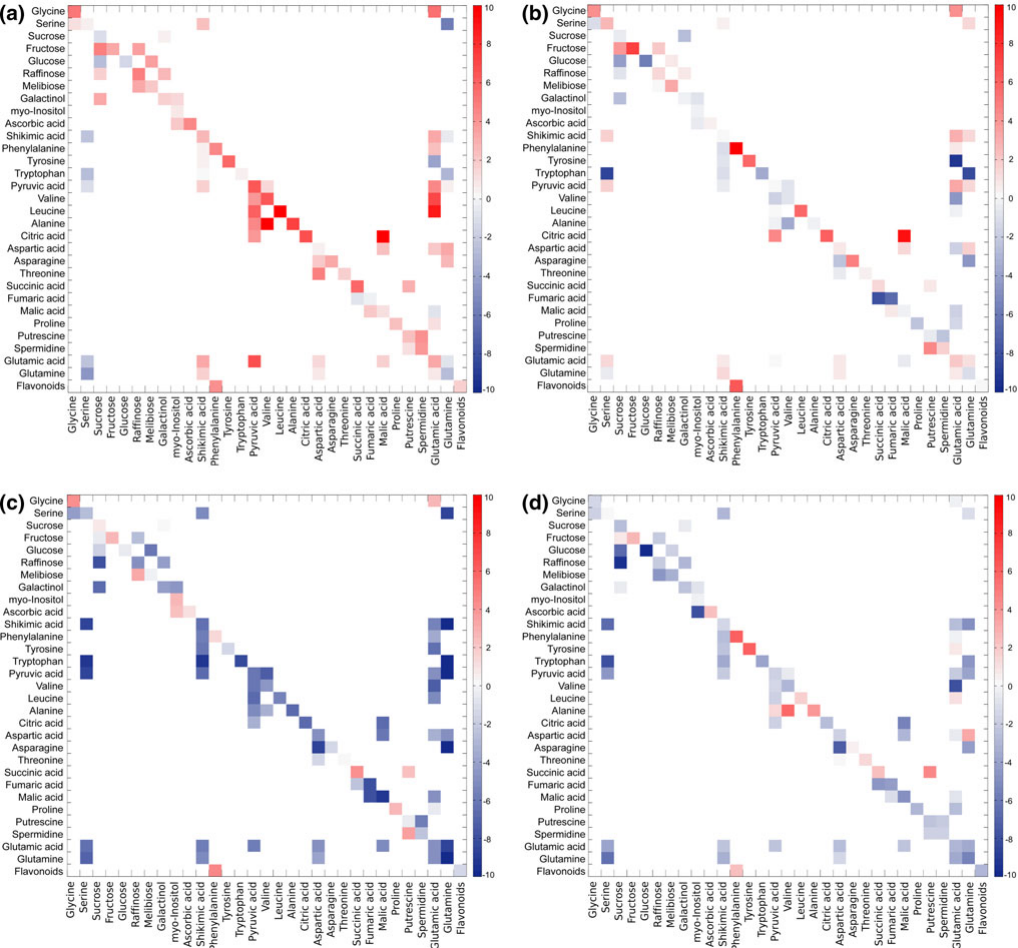
Entries of the differential Jacobian after 2d(**a**), 8d(**b**), 14d(**c**) and 18d(**d**) of cold exposure relative to the non-acclimated condition are visualized by the heat map. Red colours indicate an increase of putative metabolic interaction while blue colours indicate a decrease relative to the non-acclimated plants. Entries of the differential Jacobian matrices represent mean values of 30 calculations

The underlying stoichiometric matrix of this network was compiled for the inverse calculation of a differential Jacobian matrix using metabolomics covariance data according to (Sun and Weckwerth 2012). Metabolic states a and b were defined by time points of differentially cold acclimated plants (state a) and, as a reference state, of non-acclimated plants (state b). With reference to metabolite levels of non-acclimated plants, calculation was performed for plants after 2 days at 4 °C (Fig. 3 a), 8 days at 4 °C (Fig. 3 b), 14 days at 4 °C (Fig. 3 c), and 18 days at 4 °C (Fig. 3 d) to reveal short-term, intermediate and long-term effects of cold exposure on metabolism. Mean values of 30 calculations were built and the ratios of mean values to standard errors of calculation are given in Supplement 4.

Resulting mean values of entries of the differential Jacobian matrices indicated a progressive perturbation of metabolism during exposure to 4 °C. Interactions of soluble sugars and pyruvic acid-derived metabolites were affected strongly after 2 days at 4 °C (Fig. 3a). After 8 days, the metabolic perturbation was only of alleviated intensity (Fig. 3b) and after 14 and 18 days it became even smaller than before cold exposure (Fig. 3c, d). Relative changes of the flavonoid pool (*Flavonoids*), being induced by relative changes of its substrate pool phenylalanine, became maximal after 8 days of cold exposure (Fig. 3b) and were dampened until 18 days at 4 °C (Fig. 3c, d).

### Integration of GC–MS and LC–MS data for a comprehensive understanding of plant-environment interactions

Cold-induced reprogramming of primary metabolism in *A. thaliana* is a prominent example of plant-environment interaction. Like many previous studies, our results show that levels of various metabolites are affected significantly by low temperature. In a comprehensive analysis of primary metabolism by GC–MS technique we were able to distinguish fast from slow metabolic reactions induced by cold exposure. Contents of putative cryoprotective compounds like sucrose, galactinol and putrescine showed a fast significant increase, thus proving their involvement in the immediate response to abiotic stress. However, in contrast to previous studies where asparagine content was found to increase significantly during cold exposure (Klotke et al. 2004; Usadel et al. 2008), we found that asparagine content decreases significantly within the first two days of cold exposure. This discrepancy may be explained by the different light intensities, which were used in the experiments. In the present study, we applied a light intensity of 250 μmol m^−2^ s^−1^, which was significantly higher than in studies of (Klotke et al. 2004) and (Usadel et al. 2008) to stimulate biosynthesis of secondary metabolites. Elevated light was shown to repress the transcription of asparagine synthetase genes (Tsai and Coruzzi 1991), and may therefore explain the observed decrease in asparagine content in the present study.

**Fig. 4:**
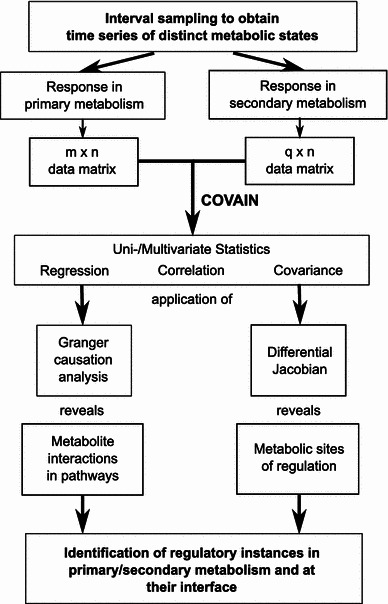
Granger causality analysis and the differential Jacobian for comprehensive analysis of GC–MS and LC–MS data sets to evaluate pathway interactions and regulatory instances in metabolism

Besides those findings, levels of tryptophan and phenylalanine were significantly decreased during the first two days of cold exposure. These aromatic amino acids (AAAs) are central metabolic precursors for synthesis of secondary metabolites (Tzin and Galili 2010). However, in contrast to primary metabolites like sugars or amino acids, plant secondary metabolites cannot be analysed by GC–MS, but LC–MS has to be applied. Although numerous approaches already provided evidence for the usefulness of a combined GC–MS and LC–MS approach (Tzin et al. 2009, 2012), these approaches were driven by the available knowledge about certain interactions between pathways of primary and secondary metabolism. The mass-to-charge (*m*/*z*) ratios, which represent the primary results of LC–MS analysis, are identified by comparison to available data bases or libraries. Although such an approach is very powerful because it allows for the simultaneous analysis of hundreds of metabolites, it is limited by the a priori knowledge about metabolic pathways. To overcome this limitation, we developed and applied statistical Granger causality analysis to unravel putative interactions of primary and secondary metabolism. Thus, shikimic acid as well as AAAs were correlated with a set of *m*/*z* ratios from LC–MS measurements which could afterwards be identified as members of the cyanidin family representing the predominant flavonoids in *A. thaliana* (Bloor and Abrahams 2002; Tohge et al. 2005). It is known from literature that accumulation of anthocyanins in leaves is stress-inducible, protecting against photoinhibitory damage caused by high irradiance (Havaux and Kloppstech 2001; Page et al. 2012). We also identified a correlation between ascorbic acid and anthocyanins, which was previously described by Page and co-workers who compared six *Arabidopsis* accessions under high light conditions (Page et al. 2012). The authors concluded from their experiments that the ability to accumulate anthocyanins in *Arabidopsis* is tuned by the status of ascorbic acid. Although we are not yet able to give a physiological interpretation of all the metabolic correlations we found by Granger causality analysis, we are now able to derive possible interactions and test them by further experimental investigation. We exemplified this by describing the metabolic network, which is represented by our GC–MS data, and expanded this network by the phenylalanine-derived synthesis of secondary metabolites identified by Granger causality analysis. Applying the inverse calculation of the differential Jacobian (Sun and Weckwerth 2012), the synthesis of secondary metabolites, termed as *Flavonoids*, were indicated to become maximally dependent on changes in phenylalanine content after 8 days of cold exposure (Fig. 3b). Here, the term *perturbation* describes the change in flavonoid content due to a relative change in phenylalanine content. Additionally, the calculation of the differential Jacobian allowed for the estimation of system behaviour after perturbation by changing environmental conditions. While entries of the differential Jacobian became positive after 2 days at 4 °C, most of them got negative after 14 days at 4 °C pointing to a change in dynamical system behaviour around the metabolic steady state. Because positive entries result from a ratio of greater than 1 this finding might indicate an increased reactivity of primary metabolites during the first 2 days of cold exposure. However, due to thermodynamic effects on enzymatic interconversions of primary metabolism at 4 °C (Nägele et al. 2012) this putative increase might be dampened and rather represents a compensation of thermodynamic effects on metabolic homeostasis than an increase in reactivity. Although this shows clearly that we cannot explicitly characterize rates of metabolic interconversions by this covariance-based approach, we are now able to detect relative changes in metabolic homeostasis due to changing environmental conditions. Additionally, deriving the Jacobian from covariance data represents a novel and convenient method to predict biochemical changes in multidimensional data sets, which is hardly feasible by classical biochemical experiments. Localizing biochemical *hot spots* from metabolomics data provides the basis for eventually understanding the perturbation dynamics in a whole metabolic network. Granger causality analysis is applied to reveal significant co-variance within the metabolic network and thereby used to extend its stoichiometric matrix (Fig. 4). As we have shown in the present study, this enables the interpretation of metabolic constitutions within a physiological context, which is fundamental for a comprehensive understanding of plant-environment interactions (Weckwerth 2011a; Nägele and Weckwerth, 2012).

## Conclusions

Based on our findings of cold-induced changes in primary and secondary metabolism of *A. thaliana*, we conclude that the identification of Granger causalities offers a novel method to comprehensively analyse GC- and LC–MS data from the same sample. Particularly, interfaces of complex biochemical networks can be characterized providing new insights in pathway regulation. The direct linkage of statistical (i.e. Granger causality analysis) with mathematical methods (differential Jacobian) is demonstrated in the present study as depicted in Fig. 4. All the described features from integration of different data sets such as GC–MS and LC–MS data to statistical methods such as Granger causality analysis and metabolic modelling using an inverse calculation of the differential Jacobian are implemented in the metabolomics toolbox COVAIN (Sun and Weckwerth 2012). The calculation of the differential Jacobian from metabolomics data provides hints to pathway regulation, however, these predictions need to be tested by classical biochemical methods. We propose the presented strategy as a fundamental concept to link genome-scale metabolic reconstruction and metabolomics data (Weckwerth 2011b). The approach can be systematically used for genotype-metabo-phenotype studies.

## Electronic supplementary material

Below is the link to the electronic supplementary material.

Supplementary material 1 (XLSX 12 kb)

Supplementary material 2 (XLSX 38 kb)

Supplementary material 3 (XLSX 440 kb)

Supplementary material 4 (XLSX 44 kb)

## References

[CR1] Bloor SJ, Abrahams S (2002). The structure of the major anthocyanin in *Arabidopsis thaliana*. Phytochemistry.

[CR2] Giersch C (2000). Mathematical modelling of metabolism. Current Opinion in Plant Biology.

[CR3] Granger C (1969). Investigating causal relations by econometric models and cross-spectral methods. Econometrica.

[CR4] Havaux M, Kloppstech K (2001). The protective functions of carotenoid and flavonoid pigments against excess visible radiation at chilling temperature investigated in *Arabidopsis npq* and *tt* mutants. Planta.

[CR5] Hoehenwarter W, van Dongen JT, Wienkoop S, Steinfath M, Hummel J, Erban A, Sulpice R, Regierer B, Kopka J, Geigenberger P, Weckwerth W (2008). A rapid approach for phenotype-screening and database independent detection of cSNP/protein polymorphism using mass accuracy precursor alignment. Proteomics.

[CR6] Klotke J, Kopka J, Gatzke N, Heyer AG (2004). Impact of soluble sugar concentrations on the acquisition of freezing tolerance in accessions of *Arabidopsis thaliana* with contrasting cold adaptation—evidence for a role of raffinose in cold acclimation. Plant, Cell and Environment.

[CR7] Kopka J, Schauer N, Krueger S, Birkemeyer C, Usadel B, Bergmuller E, Dormann P, Weckwerth W, Gibon Y, Stitt M, Willmitzer L, Fernie AR, Steinhauser D (2005). GMD@CSB.DB: the Golm Metabolome database. Bioinformatics.

[CR8] Matsuda F, Yonekura-Sakakibara K, Niida R, Kuromori T, Shinozaki K, Saito K (2009). MS/MS spectral tag-based annotation of non-targeted profile of plant secondary metabolites. The Plant Journal.

[CR9] Moing A, Aharoni A, Biais B, Rogachev I, Meir S, Brodsky L (2011). Extensive metabolic cross-talk in melon fruit revealed by spatial and developmental combinatorial metabolomics. New Phytologist.

[CR10] Morgan JA, Rhodes D (2002). Mathematical modeling of plant metabolic pathways. Metabolic Engineering.

[CR11] Nägele T, Henkel S, Hörmiller I, Sauter T, Sawodny O, Ederer M, Heyer AG (2010). Mathematical modelling of the central carbohydrate metabolism in *Arabidopsis thaliana* reveals a substantial regulatory influence of vacuolar invertase on whole plant carbon metabolism. Plant Physiology.

[CR12] Nägele T, Stutz S, Hörmiller II, Heyer AG (2012). Identification of a metabolic bottleneck for cold acclimation in *Arabidopsis thaliana*. The Plant Journal.

[CR13] Nägele T, Weckwerth W (2012). Mathematical modeling of plant metabolism—from reconstruction to prediction. Metabolites.

[CR14] Page M, Sultana N, Paszkiewicz K, Florance H, Smirnoff N (2012). The influence of ascorbate on anthocyanin accumulation during high light acclimation in *Arabidopsis thaliana*: further evidence for redox control of anthocyanin synthesis. Plant, Cell and Environment.

[CR15] Rios-Estepa R, Lange BM (2007). Experimental and mathematical approaches to modeling plant metabolic networks. Phytochemistry.

[CR16] Scherling C, Roscher C, Giavalisco P (2010). Metabolomics unravel contrasting effects of biodiversity on the performance of individual plant species. PLoS ONE.

[CR17] Shi M-Z, Xie D-Y (2010). Features of anthocyanin biosynthesis in *pap1*-*D* and wild-type *Arabidopsis thaliana* plants grown in different light intensity and culture media conditions. Planta.

[CR18] Steuer R, Kurths J, Fiehn O, Weckwerth W (2003). Observing and interpreting correlations in metabolomic networks. Bioinformatics.

[CR19] Sun X, Weckwerth W (2012). COVAIN: a toolbox for uni- and multivariate statistics, time-series and correlation network analysis and inverse estimation of the differential Jacobian from metabolomics covariance data. Metabolomics.

[CR20] Tohge T, Nishiyama Y, Hirai MY, Yano M, Nakajima J, Awazuhara M, Inoue E, Takahashi H, Goodenowe DB, Kitayama M, Noji M, Yamazaki M, Saito K (2005). Functional genomics by integrated analysis of metabolome and transcriptome of Arabidopsis plants over-expressing an MYB transcription factor. The Plant Journal.

[CR21] Tsai F, Coruzzi G (1991). Light represses transcription of asparagine synthetase genes in photosynthetic and nonphotosynthetic organs of plants. Molecular and Cellular Biology.

[CR22] Tzin V, Galili G (2010). New insights into the shikimate and aromatic amino acids biosynthesis pathways in plants. Molecular Plant.

[CR23] Tzin V, Malitsky S, Aharoni A, Galili G (2009). Expression of a bacterial bi-functional chorismate mutase/prephenate dehydratase modulates primary and secondary metabolism associated with aromatic amino acids in Arabidopsis. The Plant Journal.

[CR24] Tzin V, Malitsky S, Ben Zvi MM, Bedair M, Sumner L, Aharoni A, Galili G (2012). Expression of a bacterial feedback-insensitive 3-deoxy-d-arabino-heptulosonate 7-phosphate synthase of the shikimate pathway in Arabidopsis elucidates potential metabolic bottlenecks between primary and secondary metabolism. New Phytologist.

[CR25] Usadel B, Bläsing OE, Gibon Y, Poree F, Höhne M, Günter M, Trethewey R, Kamlage B, Poorter H, Stitt M (2008). Multilevel genomic analysis of the response of transcripts, enzyme activities and metabolites in Arabidopsis rosettes to a progressive decrease of temperature in the non-freezing range. Plant, Cell and Environment.

[CR26] Walther D, Strassburg K, Durek P, Kopka J (2010). Metabolic pathway relationships revealed by an integrative analysis of the transcriptional and metabolic temperature stress–response dynamics in yeast. OMICS.

[CR27] Waridel P, Wolfender J-L, Ndjoko K, Hobby KR, Major HJ, Hostettmann K (2001). Evaluation of quadrupole time-of-flight tandem mass spectrometry and ion-trap multiple-stage mass spectrometry for the differentiation of **C**-glycosidic flavonoid isomers. Journal of Chromatography A.

[CR28] Weckwerth W (2003). Metabolomics in systems biology. Annual Review of Plant Biology.

[CR29] Weckwerth W (2011). Green systems biology—from single genomes, proteomes and metabolomes to ecosystems research and biotechnology. Journal of Proteomics.

[CR30] Weckwerth W (2011). Unpredictability of metabolism—the key role of metabolomics science in combination with next-generation genome sequencing. Analytical and Bioanalytical Chemistry.

[CR31] Weckwerth W, Wenzel K, Fiehn O (2004). Process for the integrated extraction identification, and quantification of metabolites, proteins and RNA to reveal their co-regulation in biochemical networks. Proteomics.

[CR32] Winkel-Shirley B (2002). Biosynthesis of flavonoids and effects of stress. Current Opinion in Plant Biology.

